# Spontaneous Coronary Artery Dissection in the Setting of Marijuana: A Case Report

**DOI:** 10.7759/cureus.59284

**Published:** 2024-04-29

**Authors:** FNU Zafrullah, FNU Raheela, Farman Ali, Shumaila Zafar, Nusrat Ayoob, Abdul Majid, Abdul Subhan Talpur

**Affiliations:** 1 Interventional Cardiology, Ascension Borgess hHospital, Kalamazoo, USA; 2 Internal Medicine, Chandka Medical College, Larkana, PAK; 3 Internal Medicine, Ascension Borgess Hospital, Kalamazoo, USA; 4 Medicine, St. John Hospital and Medical Center, Detroit, USA; 5 Internal Medicine, Wahid Medical Primary Care, Brooklyn, USA; 6 Internal Medicine, Liaquat University of Medical and Health Sciences, Jamshoro, PAK; 7 Internal Medicine, United Health Services (UHS) Wilson Medical Center, Johnson, USA

**Keywords:** cardiovascular risk, smoking cessation, coronary angiography, marijuana, spontaneous coronary artery dissection

## Abstract

This case report presents a detailed examination of spontaneous coronary artery dissection (SCAD) in a 61-year-old Middle Eastern male with a history of marijuana use and essential hypertension. The patient's emergency presentation with loss of consciousness and subsequent diagnostics - including elevated troponins and distinctive electrocardiogram changes - led to the identification of extensive SCAD affecting multiple coronary arteries. The association between marijuana use and cardiovascular pathology is focal in this study, particularly considering the patient's positive test for tetrahydrocannabinol (THC) and significant smoking history. This case highlights the critical need for heightened awareness among clinicians regarding the implications of recreational marijuana use, particularly in individuals with predisposing cardiovascular risk factors. Furthermore, it illustrates the complexity of diagnosing and managing SCAD, a condition that may vary widely in its presentation and severity, necessitating a tailored approach to treatment that considers both the acute manifestations and underlying contributory factors such as substance use.

## Introduction

“Artery Dissection” is the term used to describe the detachment of the intramural layers of an artery by a hematoma, with or without the presence of an intimal tear. When this phenomenon occurs as a cardiovascular event involving the coronary arteries, it is described as spontaneous coronary artery dissection (SCAD) [1]. SCAD is a rare condition; however, 70%-80% of the reported cases were found to occur in women, and around 80% of these cases involved the left anterior descending (LAD) artery. The remaining cases involved the Right Coronary Artery (RCA) and predominantly involved men [2,3].

SCAD can clinically present over a spectrum of symptoms depending on the extent of the dissection, ranging from an asymptomatic state or with minimal symptoms like unstable angina to life-threatening conditions like ventricular arrhythmias, myocardial infarction, sudden cardiac death (SCD), or acute coronary symptoms (ACS) [4]. The occurrence of SCAD is linked to various conditions which involve states of emotional stress, physical stress (coughing, vomiting, extreme retching, etc), connective tissue disorders (Marfan syndrome and Ehler-Danlos Type IV), autoimmune conditions (Vasculitis, Systemic Lupus Erythematosus, Celiac Disease, and sarcoidosis), stimulant use (like cannabis) and most notably, pregnancy, which imposes a hemodynamic burden on the female, along with hormonal factors [5,6].

Coronary Angiography is the mainstay diagnostic tool used in SCAD. The findings may be supplemented by employing intravascular ultrasound (IVUS) and optical coherence tomography (OCT) to provide exaggerated morphological features alluding to the location and clinical assessment of SCAD. Multidetector computed tomography (MDCT), a form of non-invasive coronary angiography, is used for the follow-up evaluation of SCAD patients [7].

Marijuana is a common and widely used drug amongst all age groups. It produces a euphoric state post-consumption, attributable to the presence of cannabinoids. Δ9-Tetrahydrocannabino (THC) is the main cannabinoid in marijuana and acts on the respective receptors in various parts of the body, including the heart [8]. The effect of marijuana in causing increased heart rate has been well reported, along with the growing evidence that marijuana causes hemodynamic changes that place stress on the heart and can lead to multi-vessel SCAD, especially in patients with pre-disposing atherosclerotic risk factors such as smoking, similar to the case presented in this case report.

## Case presentation

A 61-year-old Middle Eastern male with a known history of essential hypertension was brought to the Emergency Department (ED) after being discovered unconscious by his wife in their bedroom. The initial observation by his wife included him lying on the floor with foam at the mouth, suggesting an acute neurological or cardiac event. Upon arrival at the ED, the initial assessment revealed a troponin level of 0.04, which slightly increased to 0.05 a few hours later, indicating myocardial injury but not a full-blown myocardial infarction. The electrocardiogram (EKG) documented a right bundle branch block and T-wave abnormalities in the lateral leads, which are often suggestive of ischemic changes or structural cardiac abnormalities.

Further diagnostic investigation was pursued through cardiac catheterization, which uncovered a diffuse dissection involving the mid-distal portion of the LAD artery, the posterior descending artery (PDA), the second diagonal branch of the LAD, and the first obtuse marginal branch (OM1) of the left circumflex artery. These findings confirmed the diagnosis of SCAD. The echocardiogram supported these findings showing a left ventricular ejection fraction (LVEF) between 35% and 40% and a fixed mural thrombus on the apical wall of the left ventricle, which are indicators of significant cardiac dysfunction.

**Figure 1:**
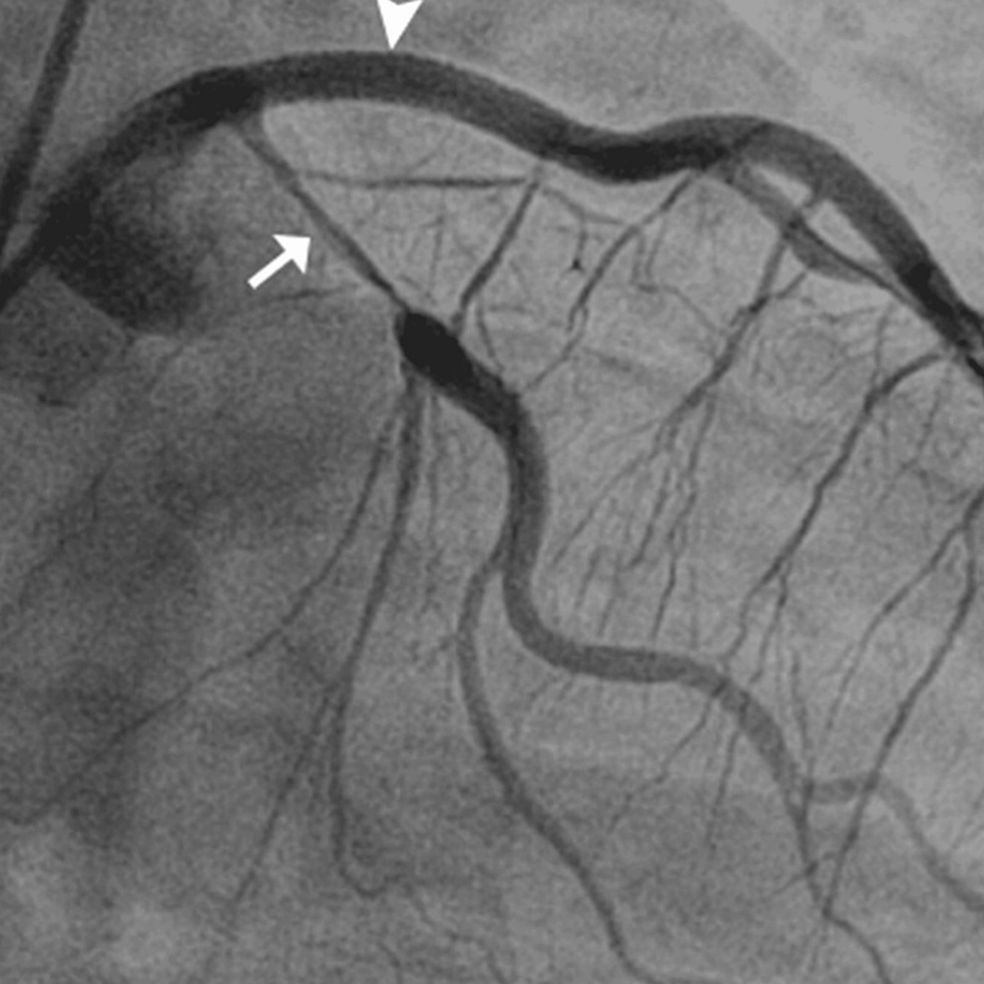
The angiographic findings showing diffuse coronary artery dissection - part 1

**Figure 2:**
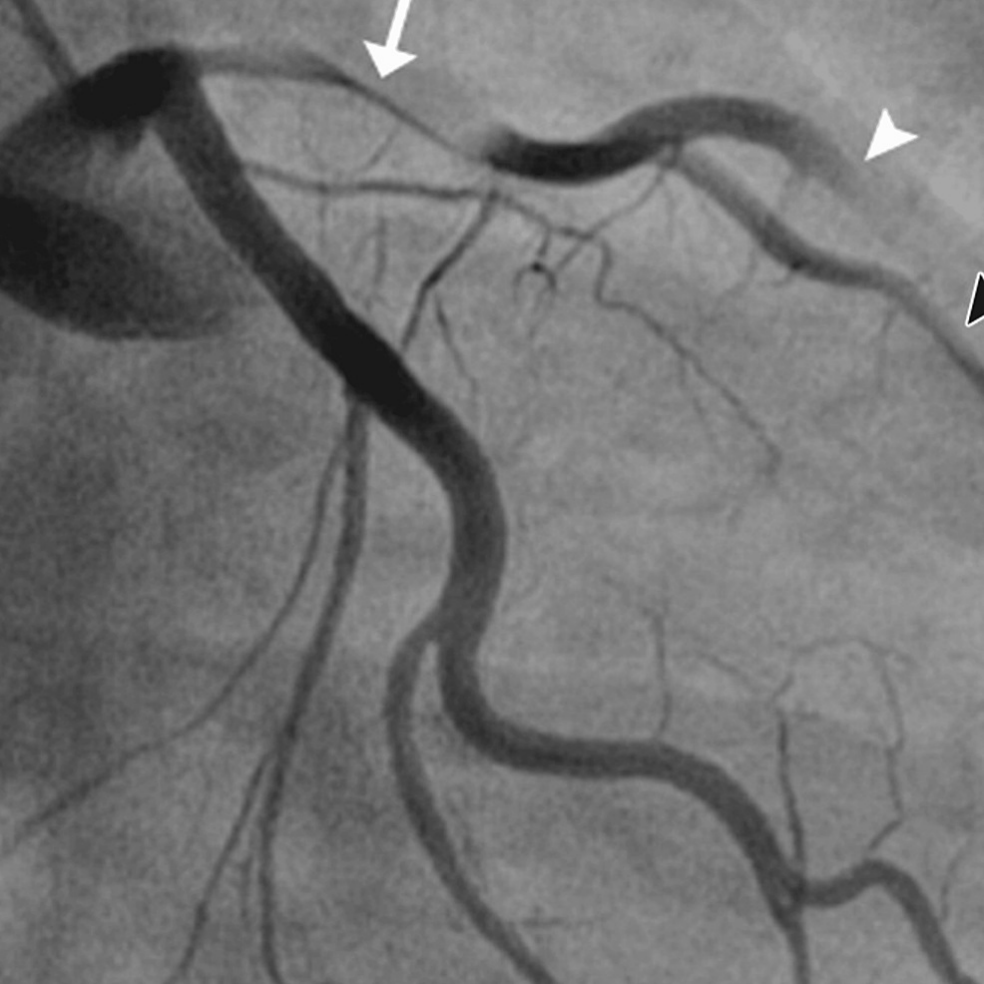
The angiographic findings showing diffuse coronary artery dissection - part 2

**Figure 3:**
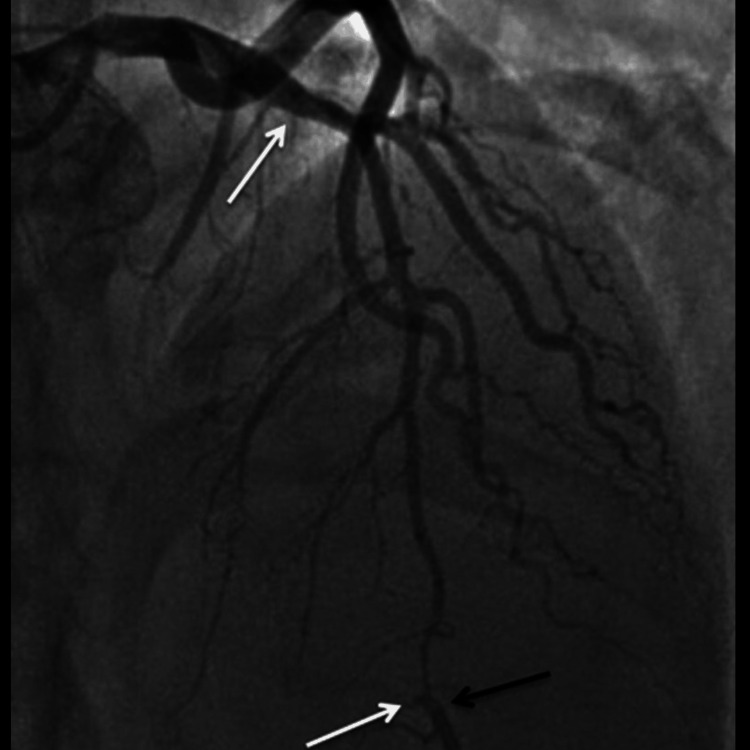
The angiographic findings showing diffuse coronary artery dissection - part 3

The patient's urine toxicology screen returned positive for Δ9-THC, indicating recent marijuana use, which could have contributed to his cardiovascular event given the known hemodynamic effects of cannabinoids. The angiographic findings illustrating the progression and extent of the coronary artery dissections are sequentially shown in Figures 1-3, providing visual confirmation of the clinical and diagnostic narrative described above.

The therapeutic approach was multifaceted, including pharmacological and monitoring strategies. He was initiated on a statin and dual antiplatelet therapy (DAPT) comprising aspirin and clopidogrel to manage his SCAD and prevent further thrombotic events. Additionally, metoprolol was prescribed to manage his cardiac workload and arrhythmic risks. Considering the left ventricular dysfunction and the presence of a mural thrombus, apixaban (Eliquis) and lisinopril were started to address the risks of thromboembolism and to support cardiac function, respectively.

Given the initial presentation of syncope, an event monitor was placed for a 16-day period to continuously assess cardiac rhythm, during which the patient predominantly demonstrated a sinus rhythm. Notably, the monitoring detected one episode of bradycardia and six supraventricular tachycardia events, which were non-life-threatening but warranted further outpatient follow-up. The patient was scheduled for regular follow-ups in the outpatient setting, emphasizing the importance of smoking cessation counseling given his substance use and its potential contribution to his cardiac condition.

## Discussion

Marijuana is a preparation of cannabis derived from the Cannabis sativa plant and used as a recreational drug for its ecstatic and psychedelic effects [9]. The active component in marijuana is THC which exerts its effects through the cannabinoid receptors type 1 and type 2 (CB-1 and CB-2). CB-1 receptors are predominantly found in hepatic, brain, muscle, and fat tissue, while CB-2 receptors are densely found in spleen and immune cells, scarcely in the peripheral tissues, and also in cardiac and smooth muscle cells, and coronary endothelial cells [10].

Marijuana has numerous physiological effects which may predispose users to SCAD. It is well documented that marijuana increases heart rate and has been implicated in the development of tachyarrhythmias with the ability to induce atrial fibrillation and premature ventricular beats, even in younger patients with little to no cardiovascular risk factors [11,12]. Importantly, marijuana raises carboxyhemoglobin levels, which ultimately leads to decreased oxygen supply. This, combined with the increased oxygen demand that cannabinoids induce in coronary vessels, places a major stress on the heart [13]. In addition, marijuana modulates blood pressure with an observed increase in supine hypertension [6]. At larger doses, it causes orthostatic hypotension due to increased blood flow to the extremities and subsequent decreased vascular resistance. Furthermore, contributions to the possible development of SCAD are further supported by the fact that marijuana has pro-coagulant effects as it increases the concentration of receptors that mediate platelet aggregation and increases the concentration of factor VII [14].

Marijuana use has been implicated in several cardiovascular events, with well-documented research on its blood pressure effects and the postulation that it may lead to multi-vessel SCAD. The cardiovascular impact of marijuana culminates in angina pectoris, myocardial infarction, tachyarrhythmias, vascular complications, and congenital heart disease. It has been studied in its role in acute coronary syndrome and MI in a study of 3,882 patients who experienced an MI found that the risk was 4.8 times more likely within the first hour after smoking marijuana [12]. In patients who suffered from an MI, marijuana use was associated with a three-fold higher increase in mortality with a higher risk in frequent marijuana users [15]. Cannabis arteritis was the term coined for the peripheral vascular changes associated with chronic Marijuana consumption [16]. Affiliation of congenital anomalies like membranous ventricular septal defects and transposition of great arteries have also been likened to paternal marijuana usage [16,17].

No treatment armamentarium has been attributed to the management of SCAD, with the most pertinent option being utilized in different scenarios. Stable patients have even responded to conservative management, while antiplatelet therapies and GPIIb/IIIa inhibitors have also shown promising results [18-20]. A case documented by Kollet et al. demonstrated complete recovery in a 35-year-old woman who was administered immunosuppressive agents (prednisone and Cytoxan) in addition to conventional medical therapy [21]. A stent implantation is risky but may be performed if the true and false lumen are correctly identified. Coronary artery bypass graft (CABG) may also be a plausible option for multi-vessel involvement [22,23].

## Conclusions

In conclusion, the correlation between SCAD and marijuana use demands extensive exploration and ongoing research to aid physicians in prompt diagnosis. Our case represents a typical presentation of SCAD managed conservatively, avoiding surgical intervention. There's a critical need for exhaustive studies to establish universally applicable treatment strategies tailored to the severity and individual patient characteristics. This is essential to ensure equitable and timely management practices across all cases, avoiding any disparities in care delivery. Further investigations into the relationship between marijuana use and SCAD are imperative for enhancing our understanding and refining clinical approaches to this condition.
